# Acidification scenario of Cox’s Bazar coast of the Bay of Bengal, Bangladesh and its influence on fish larvae abundance

**DOI:** 10.1016/j.heliyon.2023.e15855

**Published:** 2023-04-27

**Authors:** Saifuddin Rana, Md. Nazmul Hasan, Nargis Sultana, Shanur Jahedul Hasan, Shahida Arfine Shimul, Sk. Ahmad Al Nahid

**Affiliations:** a Department of Fisheries Resource Management, Faculty of Fisheries, Chattogram Veterinary and Animal Sciences University, Khulshi-4225, Chattogram, Bangladesh; b Department of Oceanography, Faculty of Marine Sciences and Fisheries, University of Chittagong, Chittagong-4331, Bangladesh; c Bangladesh Fisheries Research Institute (BFRI), Mymensingh, Bangladesh

**Keywords:** Bay of Bengal, Fish larvae, Ocean acidification, Seacarb package, Cox's Bazar Coast

## Abstract

Ocean acidification is caused mainly by atmospheric carbon dioxide stored in the ocean. Ocean acidification is considered a major threat to aquatic life, and how it influences the abundance of marine fish larvae is still unclear. This research was designed to measure the current ocean acidification scenario of the Cox's Bazar coast of the Bay of Bengal, Bangladesh, and its probable influence on the abundance of fish larvae. Three research stations were selected: Bakkhali river estuary, Naf river estuary, and Rezu Khal. Monthly sampling was done, and larvae sample was collected from the surface water column (depth: 0.5 m) using a bongo net. Water parameters such as temperature, salinity, total alkalinity, and pH were determined using laboratory protocol. The seacarb package of the R programming language was used to determine ocean acidification factors. The Bakkhali river estuary showed the highest partial carbon dioxide (143.99 ± 102.27 μatm) and the lowest pH (8.27 ± 0.21). A total of 19 larvae families were identified, and the highest larval count was found in Rezu Khal (390 larvae/1000 m^3^), while the lowest was found in the Bakkhali river (3 larvae/1000 m^3^). Clupeidae, Myctophidae, and Engraulidae comprised more than 50% of the identified larvae. Blenniidae, Carangidae, Clupeidae, Engraulidae, and Gobiidae were found in all three seasons. Most of the larvae families showed the highest mean abundance under less pCO_2._ A negative correlation was observed between larvae and acidification factors such as pCO_2_, HCO_3_^−^, and dissolved inorganic carbon (DIC). The study revealed that acidification parameters of the Cox's Bazar coast were not in an acute state for the aquatic organisms' survival, but fish larvae abundance could be declined with raises in the partial carbon dioxide. The results of this study may aid in developing a management plan for conserving Bangladesh's marine and coastal fish.

## 1 Introduction

Ocean acidification and global warming arising from rising carbon dioxide concentrations in the globe's atmosphere [1,2]. The level of acidification in the ocean ecosystem is acknowledged as a critical component in the transformation of biological systems [3]. Atmospheric carbon dioxide (CO_2_) is raised over 410 ppm, nearly 50% higher than the pre-industrial level. The increased level is unprecedented in the global geological history of the previous 55 million years [4]. The origin of this excess CO_2_ is human-caused; two significant sources are anthropogenic fossil fuel and the manufacturing industry [5]. Due to human interventions, CO_2_ levels in the atmosphere are recorded at 380 ppm, rising by 0.5% each year from 2021 [6] and 100 times quicker than the previous 650 000 years [7,8]. Since the 1950s, numerous lines of evidence, such as observations of dissolved inorganic carbon (DIC), have shown that oceans deposit around a quarter of human-induced CO_2_ discharge in the atmosphere [9]. As a result, the additional atmospheric CO_2_ deposition raises the pH and makes the ocean acidic [10].

Atmospheric CO_2_ levels have risen by 400 parts per million, and scientists predict it will reach 1000 parts per million by the end of this century [11]. As a result, by 2050, atmospheric CO_2_ levels will reach 467–555 ppm, causing surface ocean pH to drop to 7.8 on average [12]. Scientists use large-scale monitoring techniques to understand ocean chemistry and thus explore changes that occur due to external factors such as global warming [13]. Human-induced increase in CO_2_ in the aquatic environment directly impacts its fauna's physiology, survival, abundance, growth, behavior, biology, metabolism, and behavioral response [14]. Acidification is an alarming issue for the current world as it is assumed that there is a direct consequence on ocean life, such as fish, mammals, corals, and other crucial resources of marine ecosystems [15]. Climate change and ocean acidification-driven environmental changes will be the two most significant threats to marine life in the coming decades [11,16].

Larvae and juvenile stages of marine fish are much more responsive to acidity and seawater temperature fluctuations than adult stages [17]. Marine species in the early stages of their lives, including their embryo and larvae stage, may suffer due to ocean acidification is well documented in the scientific literature. For instance, compared to bivalve and gastropod larvae growing in habitats with ambient ocean pH, those growing in low pH/high pCO_2_ conditions have poorer rates of survival and growth [18]. Similarly, coral larvae and juveniles growing at low pH and high pCO_2_ levels exhibit slowed calcification rates brought on by increased carbonate ion dissolution [19]. Under acidification conditions, fish larvae may experience a range of negative consequences, including increased mortality rates, slowed growth rates, and sensory impairment [[20], [21], [22]]. Several studies show that larval fish may also be vulnerable to the impacts of ocean acidification [[23], [24], [25]]. Ocean acidification may have impacted larval growth and survival during the past 200 years, which may have led to a global drop in the number of bivalve species that are significant for both economic and ecological reasons [12,26]. Phytoplankton response to rising CO_2_ levels and changes in ocean chemistry could affect energy transfer, nutrient cycling, and carbon cycling in coastal waters [27].

Several studies reported the negative consequences of acidification's effects on aquatic organisms' physiology, cellular mechanisms, sensory perception, and ecosystem-level dynamics [18,21,28]. On the other hand, ocean acidification's effects on fish larvae abundance are still unclear due to the lack of adequate research [29]. However, some recent studies report predicts that within the next 100 years, the elevated impact of acidification on marine fish and inveterate will be visible [30,31]. Continued increases in atmospheric carbon dioxide in the future century could have significant consequences for a wide range of marine fish species [29,32].

The objective of this study was to evaluate the state of ocean acidification factors, their effects, and the relationship between those factors and the number of fish larvae along Bangladesh's Cox's Bazar coast. In the context of Bangladesh, this study on how ocean acidification affects larval abundance is the time needed. This study's findings may be helpful in the decision-making process of coastal fisheries management and conservation of the ecologically essential fishes in Bangladesh. Therefore, adequate research and continuous monitoring programs are required to determine ocean acidification's current and future state.

## 2 Materials and methods

### 2.1 Selection of the research stations

**Fig. 1:**
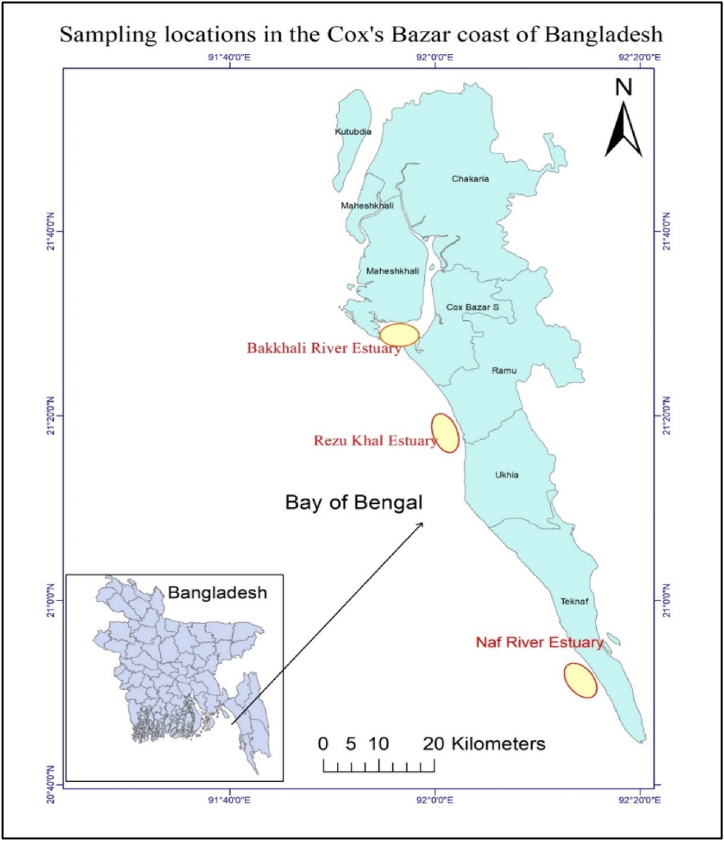
Selected sampling locations are named Bakkhali River Estuary, Naf River Estuary, and Rezu Khal on Cox's Bazar coast of the Bay of Bengal for the collection of fish larvae and water sample.

This research was performed for 12 months in three stations on Cox's Bazar Coast, Bangladesh. Three stations were selected considering river discharge and other parameters to estimate the relationship between larval abundance and ocean acidification. The areas were Bakkhali River Estuary (21.487971° N, 91.965496° S), Rezu Khal Estuary (21.296307° N, 92.034257° S), and Naf River Estuary (20.8636840° N, 92.2506240° S) (Fig. 1). The following research station map was constructed using ArcGIS (Version 10.8.1) based on the GPS coordinates.

### 2.2 Sample collection

The sampling was done monthly for one year to visualize the acidification scenario of Cox's Bazar coast in Bangladesh. Water and fish larvae samples were collected from the chosen site to evaluate the hydrological parameters of the study region and determine the number of larvae. Water samples were collected using a long-weighted tube to collect integrated samples. The larval fishes were sampled from subsurface water using a Bongo net (Diameter 60 cm, Length 2.5 m, and mesh size 500 μm). A flow meter (Model: KC Denmark A/S 23.091) was attached to the Bongo net to measure the volume of water passing through the Bongo net. The selected research stations were sampled in the surface water column (depth: 0.5 m). Samples were transported to the Aquatic Ecology Laboratory of Chattogram Veterinary & Animal Sciences University for further analysis.

### 2.3 Determination of larvae abundance and families

The larvae sample was sorted and identified using a stereomicroscope (Optika C–B3). Larvae samples were separated for the total count and counted to determine larval abundance. Counting was done three times to avoid an error. Total abundance was determined using the following formula:

• The volume of water passed in each sampling = Indicated number of revolutions × Pitch of the impeller (0.3) × Net opening area (m^2^) × 1000
• Number of larvae per 1000 m^3^= (Number of larvae in sample × 1000) / Volume of water passed

A stereomicroscope (OPTIKA ITALY C–B3) at a modest magnification (10X) was used to identify fish larvae. Fish larvae families were identified by comparing morphological features under the stereomicroscopic view with the fish larvae features described in the previously published literature [33,34]. Larvae were not distinguished based on their morphological characteristics and were categorized as unidentified.

### 2.4 Analysis of the water parameters and determination of acidification factors

Several water parameters, such as Dissolved Oxygen (DO), Temperature, pH, Salinity, and Alkalinity, were measured to define the acidification scenario of the Bay of Bengal. Dissolved oxygen (DO) and temperature were measured using an electronic probe (Model: JANEWAY-9500). The total alkalinity of collected water samples was calculated using the Gran titration method, where Phenolphthalein and Methyl Orange were used as an indicator, and salinity was measured using a Refractometer (Model: Hanna HI-96822). Seawater samples were collected in gas-tight bottles, avoiding trapping air bubbles when capping bottles to determine water pH. The electrode-probe method was used to measure pH in the laboratory (Model: Hanna HI-2211). The most widely utilized indices to forecast the acid-base concentrations in seawater were total dissolved inorganic carbon (DIC), total alkalinity (TA), H^+^ concentration, and pCO_2_. However, the current study used total alkalinity and H+ concentration. The study used the “seacarb” package of R programming, where the variables were k1 and k2 [35] Ks and K_f_ [36].

### 2.5 Statistical analysis

Pearson's product-moment correlation was run to assess the degrees and direction of the relationship among the hydrological parameter, ocean acidification variables, and larvae abundance with a 95% confidence interval. Data normality was evaluated by the Shapiro-Wilks test (p > 0.05). Data was curated, modeled, and presented using R programming, Statistical Package for the Social Sciences (SPSS version 27), and Statistical Analysis Software: John's Macintosh Project (SAS JMP version 14). Mean values of the larvae abundance were expressed with standard deviation (SD). The constrained Canonical Correspondence Analysis was done between acidification parameters, and larvae count using XLSTAT (version 2018.1). The Canonical Correspondence Analysis map allows understanding to visualize the larvae family, the sites, and the environmental variables.

## 3 Results

### 3.1 Hydrological parameters and acidification factors

The mean partial pressures of CO_2_ in three selected sampling sites were 143.99 ± 102.27, 82.94 ± 36.86, 128.72 ± 124.41 (μatm), respectively, corresponding to pH values of 8.27 ± 0.21, 8.38 ± 0.20, and 8.37 ± 0.33 in Bakkhali, Rezu Khal Estuary, and Naf Estuary (Table 1). The highest pCO_2_ and lowest pH were found in the Bakkhali River, indicating more acidic conditions than the other research stations. Table 1 shows the parameters of the seawater carbonate system for the different stations during the study period. Variables were calculated using measured Temperature (T), pH, Total Alkalinity (A_T_), and Salinity (S) and expressed with means ± SD.

**Table 1:** The calculated acidification factors from the water quality parameters in the selected research stations in the study area.

Station name	Seawater measurements				Calculated factors for acidification					
	T (°C)	pH	A_T_ (mg/L)	S (psu)	pCO_2_ (μatm)	HCO_3_^−^(μmol kg^−1^)	CO_3_^2^ (μmol kg^−1^)	DIC (μmol kg^−1^)	Ω_Aragonite_	Ω_Calcite_
Bakkhali River	28.85 ± 2.19	8.27 ± 0.21	117.75 ± 40.53	24.12 ± 6.60	143.99 ± 102.27	809.06 ± 361.52	133.84 ± 45.77	946.87 ± 385.33	2.28 ± 0.76	3.54 ± 1.11
Rezu Khal Estuary	27.65 ± 2.34	8.38 ± 0.20	104.17 ± 22.4	23.98 ± 5.64	82.94 ± 36.86	651.69 ± 157.65	138.54 ± 43.09	792.54 ± 179.58	2.34 ± 0.68	3.66 ± 1.03
Naf Estuary	28.42 ± 2.56	8.37 ± 0.33	113.25 ± 39.31	23.58 ± 7.99	128.72 ± 124.41	750.82 ± 370.36	137.75 ± 33.66	891.93 ± 387.43	2.35 ± 0.55	3.70 ± 0.86

* Values were presented with the Mean ± SD.

### 3.2 Variation of total fish larvae and their family abundance in response to pCO_2_

**Fig. 2:**
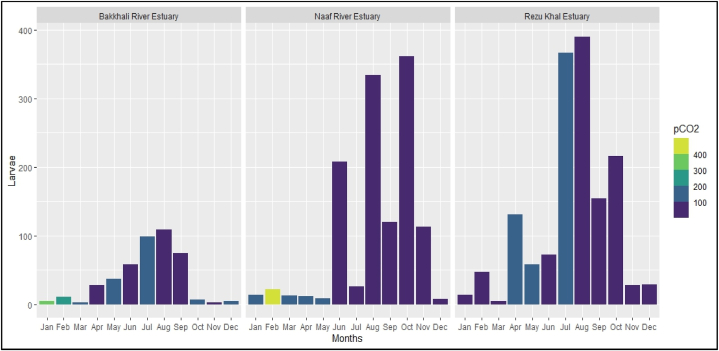
Monthly and station-wise variation of larvae abundance (per 1000 m^3^) and response to the partial pressure of CO_2_. The X-axis of the figure represents the sampling month, while the Y-axis represents the larvae abundance's mean value. The larvae count in Bakkhali, Rezu Khal and Naf river estuary were 13.5 ± 20.5, 13.6 ± 70.8, and 41.48 ± 84.0, respectively. The staked color of the bar shows the level of pCO_2_. (For interpretation of the references to color in this figure legend, the reader is referred to the Web version of this article.)

In August, the Rezu Khal had the highest larval count (390 larvae/1000 m^3^), while the Bakkhali river had the lowest (3 larvae/1000 m^3^). At Naf River, both the highest (464 μatm) and lowest (5 μatm) pCO_2_ levels were recorded in February and August, respectively (Fig. 2). The Rezu Khal and Naf estuaries had higher larval abundance than the Bakkhali river estuary. Fig. 2 shows that the larval abundance was decreased in most cases where pCO_2_ was high.

**Fig. 3:**
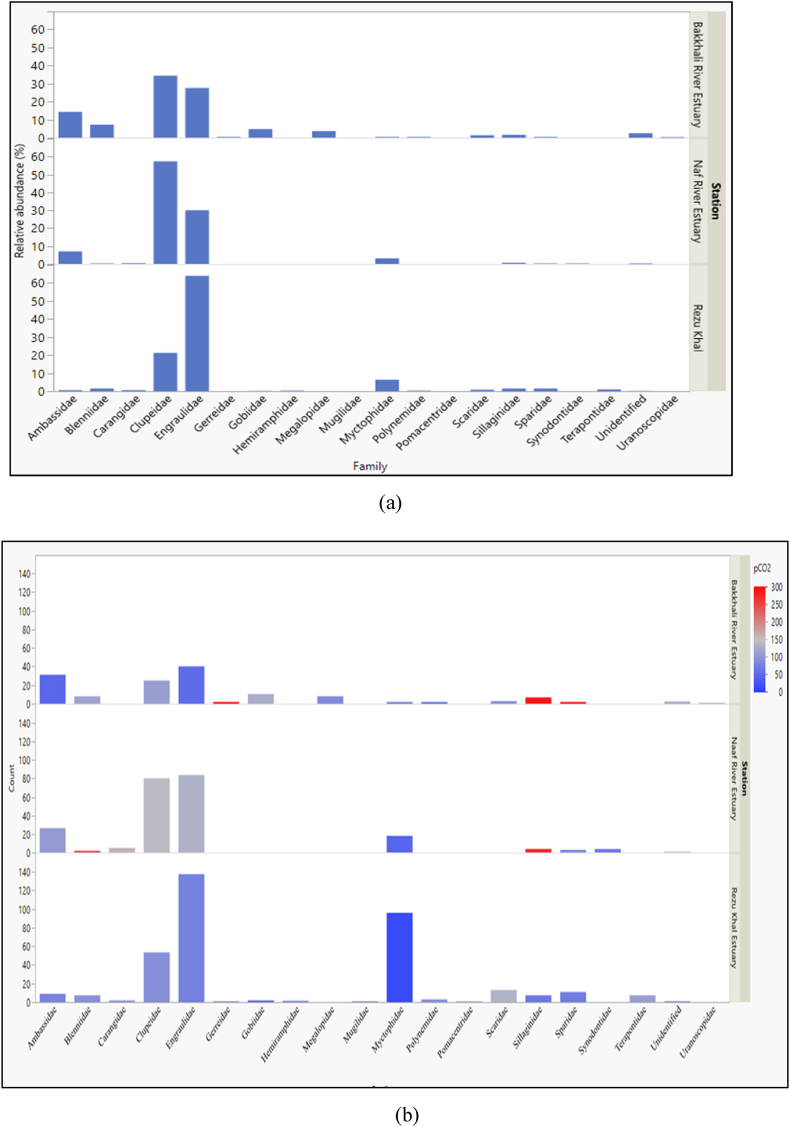
**(a)** Family-wise variation of relative abundance of larvae at the three stations. **(b)** Family-wise variation of larvae abundance (per 1000 m^3^) and response to the partial pressure of CO_2_.

Nineteen (19) larvae families were identified, and Clupeidae, Myctophidae, and Engraulidae shared more than 50% of the total identified larvae count (Fig. 3a). The availability of a single larvae family over the year depends on several parameters. The larvae family, such as Blenniidae, Carangidae, Clupeidae, Engraulidae, and Gobiidae, were present in all three seasons pre-monsoon, monsoon, and post-monsoon (Table 2).

**Table 2:** Season-wise variation of larvae family for the three sampling locations.

	Bakkhali Estuary			Rezu Khal			Naf River Estuary		
Family	PM	M	PoM	PM	M	PoM	PM	M	PoM
Ambassidae		*			*			*	*
Blenniidae	*	*			*	*		*	*
Carangidae				*	*	*	*		
Clupeidae	*		*	*	*	*	*		*
Engraulidae		*		*	*	*	*	*	*
Gerreidae			*	*					
Gobiidae	*	*				*			
Hemiramphidae				*		*			
Megalopidae		*							
Mugilidae				*					
Myctophidae		*			*			*	*
Polynemidae		*			*				
Pomacentridae					*				
Scaridae		*			*				
Sillaginidae			*			*			*
Sparidae			*			*			*
Synodontidae								*	
Terapontidae					*	*			
Uranoscopidae		*							
Unidentified	*	*	*	*	*			*	*

*PM = Pre-Moonson, M = Monsoon, PoM = Post-Monsoon.

Most larvae families showed the highest mean abundance under less pCO_2_ (Fig. 3b). Several larvae families, such as Blennidae, Sillaginidae, Scaridae, and Gerreidae, were sustained at comparatively higher acidic conditions than others. Larvae whose families were not identified based on morphological attributes were categorized as unidentified.

### 3.3 Relationship between acidification factors and larvae abundance

**Fig. 4:**
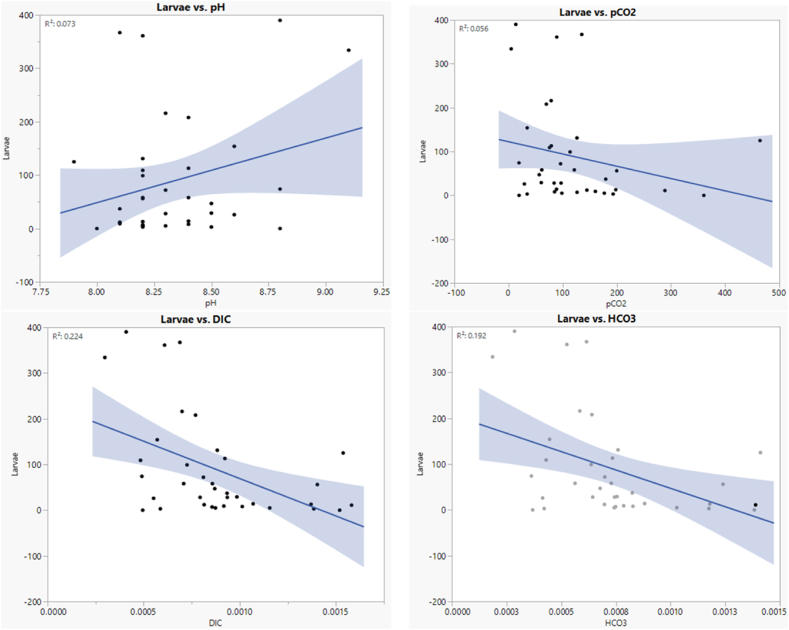
Correlation matrix plots for the understanding relationship between the total larvae count (1000 m^3^) and ocean acidification parameters such as dissolved inorganic carbon (DIC), pH, partial carbon dioxide (pCO_2_), and bi-carbonate (95% confidence intervals).

A correlation between acidification factors and larvae abundance was done to visualize the effects of acidification on the fish larvae’ abundance. Fig. 4 shows no significant correlation between mean larvae abundance and acidification parameters (p > 0.05). It showed a negative relationship for the larvae abundance with dissolved inorganic carbon (DIC), pCO_2_, and bicarbonate ion concentration but a positive relationship with pH (*p* > 0.05). The coefficient of determination value, the R^2^ between mean larvae count and dissolved inorganic carbon, was found to be 0.224, 0.073 for pH and larvae count, 0.056 for pCO_2_ and larvae count, and 0.192 for HCO_3_^−^ and larvae. The relationships show that increased acidification variables, such as DIC, pCO_2_, and HCO_3_^−^ lowered the number of larvae.

### 3.4 Relationship between the acidification parameters and identified larvae family

**Fig. 5:**
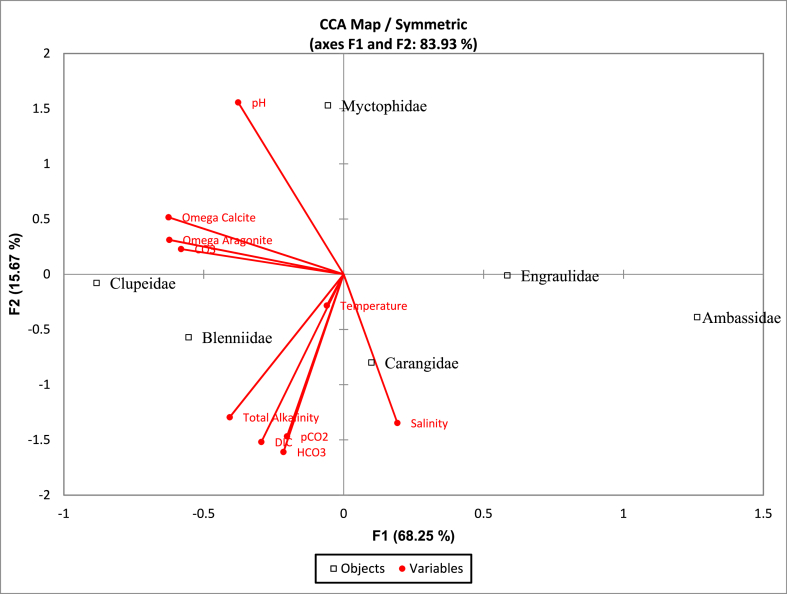
Canonical correspondence analysis bi-plot of the environmental variables where red lines were denoted as vectors and squares represent the abundance of the specific larvae family. (For interpretation of the references to color in this figure legend, the reader is referred to the Web version of this article.)

Canonical Correspondence Analysis (CCA) provides insight into the relationship between the acidification parameters and identified significant larvae families in the study area. The six most abundant larvae families out of 19 were included in the analysis as they contributed the major portion of the total larvae counts. The plot was divided into four axes, and the eigenvalues and vector length indicate the significance of variables in the CCA. The analysis was significant overall at a 95% confidence interval (permutation test for CCA). Fig. 5 shows that all the environmental variables are important for the larvae abundance in the study area, but the temperature has less effect on the larvae family. The abundance of the Carangidae family was highly correlated with salinity, and Blenniidae, Clupeidae, and Myctophidae are likely sensitive to total alkalinity, carbonate ion, and pH, respectively.

## 4 Discussion

Acidification parameters such as partial carbon dioxide, dissolved inorganic carbon, and calcite saturation rate are considered established methods for determining the acidification of a water body [37]. According to the values of the acidification parameters described in Table 1, the condition of acidification in the study area is not acute for the organisms such as dissolved inorganic carbon (DIC) in all stations was less than 1000 μmol/kg, which is typically comprised of CO_3_^2−^ and HCO^3−^and is considered a key component of salt water and an essential indicator for ocean acidification. DIC is less than 2000 μmol/kg and is suitable for organisms [38]. A study suggests that high DIC concentrations may lead to reduced accretion and growth of coral reefs [39].

The aquatic organisms become agitated when the aragonite saturation level falls below 3, and at a level below 1, the organism's shells begin to dissolve [40]. In all the stations of the present study, aragonite saturation values were greater than 1. Undersaturated salt water (Ω_calcite_ <1) is regarded to be corrosive to calcifying organisms. The ability of seawater to erode the CaCO_3_ shells and skeletons of marine creatures is measured by its saturation level for CaCO_3_ [41]. In this context, the study documented calcite values greater than 3, indicating sufficient calcite availability for calcifying organisms. The study's findings showed the appropriate states of the acidification parameters in Cox's Bazar coast for aquatic organisms compared to the above-cited reference values of acidification factors.

The present study measured temperature, pH, total alkalinity, and salinity at 22.6–32.0 °C, 7.9–9.1, 52–185 mg/L, and 8–34 psu, respectively (Table 1). The global acidification scenario predicted that the Bay of Bengal pH level would reduce to 7.8 by 2095 [42], but the present study identified less than the predicted values. Temperature and salinity in the Bay of Bengal ranged between 23 and 32 °C and 22–34 psu, also agreed with the present study's identified ranges [43]. According to Fig. 5, environmental parameters and acidification factors have influenced the larvae abundance, as salinity had the strongest influence on the family Carangidae. Carbonate, pH, and alkalinity affect the abundance of Blenniidae, Clupeidae, and Myctophidae. No precise information is available on the identified dominant larval family species along the Cox's Bazar coast. However, there are some studies which were identified the most abundant species of dominant families in several locations, such as *Spratelloides delicatulus* (Clupeidae), *Omobranchus puctatus* (Blenniidae), and *Gerres oyena* (Gerreidae), were the most abundant in Mabahiss Bay, Egypt [44]. The abundance of fish larvae varies with different factors, such as the spawning season, food availability, and environmental parameters [45]. The study's findings also indicate the influence of environmental parameters and acidification factors on fish larvae abundance.

According to Fig. 4, the present study found a negative relationship between (pCO_2_) and larvae abundance, which relates to the findings described in Fig. 2, Fig. 3. A study also found a negative relationship between pCO_2_ and larvae abundance and stated it could be due to the difference in the pCO_2_ between their body fluid and ambient medium, as aquatic organisms are very sensitive to environmental CO_2_ [46]. Some research has been conducted on the effects of elevated partial pressure on fish larvae, such as the effects of elevated pCO_2_ on the embryonic developmental phase [47], larval growth and survival [15], and tissue and organ health [48]. Larval disorders, malformations, organ failure, and stunted growth in some marine fish species have been found due to the raises of pCO_2_ [49]. Continuous monitoring of ocean acidification factors is required to follow up on the effects of acidification on fish larvae because it is still unknown whether the levels of ocean acidification that may occur over the next 100 years will negatively affect the early life-history traits of marine fish [46].

The study found an influence of the acidification parameters on the larvae abundance of the Cox's Bazar coast of the Bay of Bengal. Canonical corresponding analysis bi-plot (Fig. 5) identified the water quality parameters and acidification factors on the mean abundance of the larvae for the identified families, and a significant influence of the parameters was observed (p < 0.05). The correlation matrix (Fig. 4) also revealed the influence of the parameters on the larvae abundance. As the value of the pCO_2_ and other acidification factors were within the tolerable limits (Table 1) for the fish larvae, the elevated effect of the acidification on larvae abundance was not observed in the study area. Based on the mean values of the acidification factors, the study indicates the appropriate state for the aquatic organisms, but meanwhile, monthly-based analysis (Fig. 2) showed negative influences of pCO_2_ on the larvae abundance. As acidification is an alarming issue for the globe, and scientists predict it will rise over time, the study suggests continuous monitoring of the acidification factors in the Bay of Bengal should strictly monitor the acidification parameters to understand the scenario of acidification at Cox's Bazar coast of the Bay of Bengal.

## 5 Conclusion

This study addressed the present acidification state of the Cox's Bazar coast of the Bay of Bengal, Bangladesh, and its influence on the abundance of fish larvae. An inverse relationship was observed between the fish abundance of larvae and the acidification components, suggesting that acidification negatively influences the larvae’ abundance. Environmental parameters, especially pCO_2_, influenced the larvae abundance in the study area. The acidification scenario of the study sites should be continued or extended for a long period to get more precise results. Monitoring the larvae abundance and acidification of the Cox's Bazar coast will enable proper visualization of the effects of acidification on the fish larvae. This study's findings may be helpful in the decision-making process for coastal fisheries management and conservation of the ecologically essential fishes in Bangladesh.

## Author contribution statement

Saifuddin Rana: Conceived and designed the experiments; Performed the experiments; Analyzed and interpreted the data; Wrote the paper.

Md. Nazmul Hasan; Nargis Sultana; Shanur Jahedul Hasan; Shahida Arfine Shimul: Performed the experiments; Contributed reagents, materials, analysis tools or data.

Sk. Ahmad Al Nahid: Conceived and designed the experiments; Performed the experiments; Analyzed and interpreted the data.

## Data availability statement

Data will be made available on request.

## Funding statement

This research was supported by 10.13039/501100008229Bangladesh Fisheries Research Institute, Bangladesh.

## Declaration of competing interest

The authors declare that they have no known competing financial interests or personal relationships that could have appeared to influence the work reported in this paper.
